# The GRADE System for Rating Clinical Guidelines

**DOI:** 10.1371/journal.pmed.1000094

**Published:** 2009-09-15

**Authors:** Brian P. Kavanagh

**Affiliations:** 1 Departments of Critical Care Medicine and Anesthesia, Hospital for Sick Children, Toronto, Canada; 2 Program in Physiology and Experimental Medicine, The Research Institute, Hospital for Sick Children, Toronto, Canada; 3 Departments of Anesthesia, Medicine & Physiology, University of Toronto, Toronto, Canada

## Abstract

Brian Kavanagh critiques the GRADE system of grading guidelines, arguing that even though it has evolved through the Evidence-Based Medicine movement, there is no evidence that GRADE itself is reliable.

## Introduction

Professional medical groups commonly issue clinical practice guidelines. Such guidelines are traditionally the result of consensus conferences or expert panels and represent attempts to synthesize—from the best available evidence and expertise—practical guidance on the best possible care. Beyond issuing a guideline, many organizations have felt the need to provide a grading of each guideline's quality, thereby conveying to the reader a sense of the confidence that might be placed in it. This article addresses only the grading of guidelines, not their use or development.

The idea that evidence in the medical literature should be graded was initially proposed in publications from McMaster University [1]–[3], with the idea of categorizing individual studies into grades of reliability ranging from randomized controlled trials (most reliable) to case reports with expert opinion (least reliable). Grading of guidelines followed, but this has been besieged with problems. To give one example, a guideline by Ferraris and colleagues gave the use of aprotonin during high-risk cardiac surgery a “high-grade” recommendation [4], but this intervention was subsequently shown to increase mortality [5].

The pursuit of better approaches to grading guidelines has resulted in GRADE (Grades of Recommendation Assessment, Development and Evaluation), introduced in 2004 [6]. GRADE has been adopted “unchanged or with only minor modifications” by national and international professional medical societies, health-related branches of government, health care regulatory bodies, and UpToDate, an on-line medical resource that is accessed by trainees and physicians in most US academic medical centers (Box 1) [7],[8].

**Box 1. Organizations That Have Adopted the Grade System**

Agency for Healthcare Research and Quality (USA)

Agenzia Sanitaria Regionale (Italy)

American College of Chest Physicians (USA)

American College of Physicians (USA)

American Thoracic Society (USA)

Ärztliches Zentrum für Qualität in der Medizin (Germany)

British Medical Journal (United Kingdom)

BMJ Clinical Evidence (United Kingdom)

COMPUS at The Canadian Agency for Drugs and Technologies in Health (Canada)

The Cochrane Collaboration (International)

EMB Guidelines (Finland/International)

The Endocrine Society (USA)

European Respiratory Society (Europe)

European Society of Thoracic Surgeons (International)

Evidence-based Nursing Südtirol (Italy)

German Center for Evidence-based Nursing “sapere aude” (Germany)

Infectious Diseases Society of America (USA)

Japanese Society for Temporomandibular Joint (Japan)

Journal of Infection in Developing Countries (International)

Kidney Disease: Improving Global Outcome (International)

Ministry for Health and Long-Term Care, Ontario (Canada)

National Board of Health and Welfare (Sweden)

National Institute for Health and Clinical Excellence (United Kingdom)

Norwegian Knowledge Centre for the Health Services (Norway)

Polish Institute for EBM (Poland)

Society for Critical Care Medicine (USA)

Society for Vascular Surgery (USA)

Spanish Society for Family and Community Medicine (Spain)

Surviving Sepsis Campaign (International)

University of Pennsylvania Health System Center for Evidence-Based Practice (USA)

UpToDate (USA)

World Health Organization (International)

The developers of the GRADE system emphasized consistency in the rating of guidelines, as well as a wish to incorporate, and distinguish between, the “strength” of each guideline and the “quality” of the underlying studies (i.e., evidence) upon which it is based. Yet there is a central paradox: while GRADE has evolved through the evidence-based medicine movement, there is no evidence that GRADE itself is reliable.

### Are Different Guidelines Externally Consistent?

GRADE is one of several different systems for grading clinical evidence and creating clinical practice guidelines based on this underlying evidence. How do these systems compare with each other?

Atkins and colleagues, from the GRADE Working Group, compared six different systems (Box 2) [9]. Twelve assessors independently evaluated each system on the basis of 12 criteria to assess the “sensibility” (overall usefulness) of the different approaches. There was poor agreement between them. In the absence of a proven gold standard, such disagreement signals concern about the inherent validity of any of these grading systems. Commenting on this lack of agreement, the authors wrote that a new system—GRADE—could overcome the problems [9].

**Box 2. Systems for Grading Evidence and Issuing Guidelines Based on the Evidence**

Atkins and colleagues compared the following six systems [6]:

- The American College of Chest Physicians Evidence-Based Guidelines (http://www.chestnet.org/education/hsp/guidelinesProducts.php)
- Australian National Health and Medical Research Council Guidelines (http://www.nhmrc.gov.au/guidelines/index.htm)
- Oxford Centre for Evidence-Based Medicine (http://www.cebm.net/)
- Scottish Intercollegiate Guidelines Network (http://www.sign.ac.uk/)
- US Preventive Services Task Force Recommendations (http://www.ahrq.gov/CLINIC/uspstfix.htm#Recommendations)
- US Task Force on Community Preventive Services Recommendations (http://www.thecommunityguide.org/about/findings.html)

But the example of the Surviving Sepsis Campaign (SSC), an important attempt to produce guidelines to improve the care of patients with sepsis or septic shock, suggests that GRADE has not overcome these problems (see Boxes 3 and 4) [10]–[11]. The endorsement of the SSC by many influential organizations underscores its importance [10]–[11]. Nonetheless, the SSC illustrates some of the important difficulties with grading in general and with the GRADE system in particular. There are three reasons why I focus here on the SSC. First, sepsis encompasses all medical and surgical specialties, accounts for over 500,000 emergency visits per year in North America alone [12], and when accompanied by shock has a mortality of over 50% [13]. Second, the SSC may have significant impact: some believe that incorporating the SSC guidelines could save up to 100,000 lives in an 18-mo interval [14]. Third, the SSC is the best known source of advice on managing sepsis and all of its recommendations carry a grading. Finally, because the SSC published two documents 4 y apart (in 2004 and 2008 [10]–[11]), it presents a unique opportunity to compare interval changes. I focus only on grading (Boxes 3 and 4), not on the controversies surrounding the SSC [15], and I do not express support for—or criticism of—any of its recommendations.

**Box 3. Antibiotic Use in Sepsis**

In 2004 the SSC guidelines recommended that for serious sepsis, intravenous antibiotic therapy should be rapidly instituted [10]; this guideline was given a grade “E.” The grading system that was in use in 2004 was adopted from Sackett's 1989 description [3]: in both cases an “E” grade corresponded to a recommendation that was supported by so-called level IV or V evidence (nonrandomized, historical controls, uncontrolled studies, expert opinion)—the lowest levels possible [10].

In 2008, the SSC issued almost the identical recommendation but this time assigned to it a grade of 1B (if shock is present) and 1D (if shock is absent), where grade 1 corresponds to a “strong” [7] recommendation [11]. Three studies published between 2004 and 2008 (none of them randomized controlled trials) supported the idea that early antibiotics reduced mortality in sepsis [45]–[47], exactly the same conclusions reached by at least six others published before 2004 [48]–[53]. Although all the studies indicated that antibiotic delay has an adverse effect, they told the clinician nothing that was new: once the need for an antibiotic is confirmed, the sooner it is administered the better. Thus it is unclear why the grading went from grade E in 2004 to grade 1B or 1D in 2004. Was the different grading simply due to the use of a different grading system in these two different years? It seems improbable that two systems describing the validity of a recommendation could arrive at such discordant conclusions. While it is easy to see how the recommendation received a meritorious commendation in 2008 [11], it is difficult to see how it did not in 2004 [10].

**Box 4. Ventilation in Acute Respiratory Distress Syndrome**

In 2004 the SSC guidelines recommended that levels of positive end-expiratory pressure (PEEP) should be set to prevent lung collapse at expiration [10]. Although most clinicians use PEEP, almost none would be able to quantify lung collapse at the end of expiration, given that atelectasis is seldom quantified. Nonetheless, the grade in 2004 was “E” (i.e., very poor) [10]. In 2008, a virtually identical recommendation received a grade of “1” (i.e., strong) [11]. The results of three randomized controlled trials examining the effect of PEEP in acute respiratory distress syndrome (ARDS) [54]–[56] were made available before the 2008 SSC conference [11]. But none of the trials analyzed PEEP and collapse in end-expiration; rather they addressed higher versus lower levels of PEEP, and broadly showed that as tested, PEEP made little or no difference to outcome [54]–[56]. Thus, there is no rationale as to how either grading was arrived at, and no basis for the difference in grading from 2004 to 2008.

### Is GRADE Internally Consistent?

#### Inter-rater agreement of GRADE

In 2005, the GRADE working group—all experts who themselves developed the GRADE system—published a pilot study of the system [16]. The study found that the kappa value (i.e., the inter-rater agreement beyond chance) for 12 judgments about the quality of evidence was very low (mean κ = 0.27; κ<0 for four judgments). The authors stated that “with discussion” they were able to considerably improve their system, but provided no supportive data. Furthermore, the presentation of GRADE that had been published a year earlier in 2004 contains neither assessment of reliability, agreement, nor proof of usefulness [6].

#### GRADE experts versus content experts

Comparing expert opinion on sepsis with the result of the GRADE process further suggests that GRADE lacks internal consistency.

First, glucose control in the critically ill is a complex issue [17]. Recent clinical data suggest no benefit to widespread application of “tight” glucose control (i.e., intensive insulin therapy) in most intensive care unit (ICU) patients [18]–[21]. Brunkhorst and colleagues state that intensive insulin therapy has “no measurable consistent benefit in critically ill patients in a medical ICU regardless of whether the patients have severe sepsis and that such therapy increases the risk of hypoglycemic episodes” [18]. Yet the senior author of that report [18], Konrad Reinhart, is a coauthor of the SSC guidelines that gave a grade 1 ranking (strong recommendation) for “moderate” glucose control and a grade 2 endorsement (a suggestion) for “tight” glucose control [11]. No evidence exists for moderate glucose control in this context, whereas the value of tight control was supported by one single-centre randomized controlled trial (RCT) [22] and opposed by four others [18],[20]–[21],[23]. Since the 2008 SSC forum [11], the largest multicentre study, the NICE-Sugar trial, reported that tight glucose control increased ICU mortality by 2.6% (OR 1.14) [24].

Second, the SSC strongly recommends (i.e., grade 1) specific resuscitation targets (blood pressure, urine output, central venous pressure, central venous oxygenation) [11], on the basis of the protocol of a commonly cited single-centre study [25]. In a different forum, the SSC states: “It is impossible to determine from the study which particular facet of the protocol was beneficial for the patients, so the protocol as a whole must be recommended” [26]. But there is considerable debate about the usefulness of this protocol—two ongoing studies are examining if the protocol is effective [27]–[28]. One of these studies is led by Derek Angus, an author of the SSC guidelines [11]. Thus, I see an inconsistency in a grading system where the most authoritative expert in the SSC panel is investigating if the protocol is useful versus the aggregate panel decision concluding a strong recommendation that it should be used [11].

### Is GRADE Inherently Logical?

#### Strength of recommendation and quality of evidence

GRADE provides an expression of the strength of the recommendation and also provides a rating on the quality of the evidence upon which the recommendation is based. In terms of strength, GRADE considers evidence to be “strong” or “weak.” The GRADE group considers strength to reflect “the degree of confidence that the desirable effects of adherence to a recommendation outweigh the undesirable effects” [7]. This component makes sense, but less so when the strength of the recommendation is dissociated from its foundation (i.e., the quality of the evidence that underpins the recommendation). The group emphasizes the importance of making this dissociation: “Separating the judgments regarding the quality of evidence from judgments about the strength of recommendations is a critical and defining feature of this new grading system” [7]. One can envision having “high-quality” knowledge that points to a small effect (high quality, low strength). The converse, low quality knowledge that yields a high-strength recommendation seems implausible, other than perhaps the avoidance of substances such as potent toxins.

#### Combining incommensurate elements

Another problem is the “leveling” process proposed to determine the quality of the evidence. GRADE ranks the quality of evidence on the basis of the type of study, “quality” issues (e.g., blinding, follow-up, sparseness of data), consistency, directness (generalizability), and effect size. The graders are instructed to raise or lower the level of quality and trade off, for example, the presence of sparse data against demonstration of a dose-response effect [6]; of course these are fundamentally different and can therefore be neither added nor subtracted.

### GRADE Has Not Been Validated

The basis for the GRADE system is articulated in several publications [6]–[7],[9],[16],[29]–[31], but none contains supportive data, proof, or logical argument for the system. Rather, there is extensive reference to other papers written largely by the same group but with no data (except a very low kappa value for inter-observer agreement) [16]. Thus, there is no literature-based proof of the validity of the GRADE system; indeed using approaches for appraising evidence proposed by the Evidence-Based Medicine Working Group [32], I would conclude that there is little basis for GRADE.

The GRADE documents suggested that strong recommendations should require little debate and would be implemented in most circumstances [7],[29]. At first glance, this may seem reasonable but there could be unanticipated consequences, such as stifling debate about many important topics, with the result that there is less thought and less research on that topic. High-level recommendations using other grading systems strongly advocated use of beta-blockade (class I, IIa) [33]–[34] and aprotinin (class 1a) [4] in specific surgical populations. But assuming that the subsequent RCTs were appropriately conducted [5],[35], the original high-level recommendations were clearly misguided [4],[33]–[34]. A major concern about any grading system is that if enshrined, potentially life-saving prospective studies might not be permitted by research ethics boards on the basis that because a guideline has been assigned a “confident” grading, equipoise does not exist.

### Popularity and Uptake

The GRADE system has been adopted as is, or with minor modifications, by a large number of professional, statutory, and medically related governance organizations (Box 1). It is hard to understand why so many organizations, many of them leading regulatory or professional groups, would adopt a system that has no proof of effectiveness and has demonstrated inconsistency [16]. There are several possible reasons for its popularity: (1) a perceived need to regulate and reduce “unnecessary” and potentially harmful variation in health care [36]; (2) GRADE uses attractive language (such as “clarity,” “consistency,” “helpfulness,” and “rigor”) [6],[37]–[38]; (3) the attraction of the promise of clinical excellence being obtainable through such a system; (4) influential bodies may adopt GRADE in order not to be left behind what some view as a “state-of-the-art” scientific advance.

### GRADE: Potential for Bias

The SSC describes in detail how members of the GRADE group interacted with the sepsis experts and influenced the grading decisions [11]. But it is not clear to me why the GRADE group needed to be involved at all in the grading decisions given that all the SSC members are experts. Given also that the GRADE criteria are conveyed as “explicit and clear” [6], there should be little need for intensive methodological consultation from the GRADE group when experts produce guidelines. While grading experts might be helpful to explain technical elements of grading, the above scenario raises the possibility of the grading process shaping the medical message.

### GRADE: Implications for Practice and Policy

The GRADE group writes that for clinicians, strong recommendations should be seen as a quality criterion or performance indicator, and for policy makers, be adopted as policy [7]. There are similar efforts underway to synthesize studies and implement practice guidelines in several countries, including the UK and the US [39]–[41]. But knowing which studies and guidelines are best (or are valid) [42] is not straightforward—high-grade recommendations (such as [4],[33]–[34]) have been later proved wrong [5],[35].

It is not clear that the opinion of a conscientious, judicious, well-educated, and experienced clinician would necessarily be inferior to a systemized opinion, such as GRADE, especially if GRADE is not valid. Conferring a “strong” rating upon a guideline will constitute a major deterrent to a clinician considering an alternative clinical route, particularly if GRADE recommendations were to be adopted as a policy by regulatory bodies [7]. Indeed warnings have been issued about proposals to convert guidelines into law [43]–[44].

### What Should Replace GRADE?

A key question that arises when a system is questioned is: what is the alternative? There is a very good alternative to using the GRADE system to rate clinical guidelines: clinicians and organizations should use published guidelines while considering the clinical context, the credentials, and any conflicts of interest among the authors, as well as the expertise, experience, and education of the practitioner. If in the future a guideline grading system is shown to improve outcome and is without harm, it could usefully be incorporated into clinical practice.
